# MR tissue phase mapping reveals reduced left ventricular velocities in patients with myocardial scar

**DOI:** 10.1186/1532-429X-18-S1-P116

**Published:** 2016-01-27

**Authors:** Amir Rahsepar, Amita Goyal, James C Carr, Michael Markl, Jeremy D Collins

**Affiliations:** Radiology, Northwestern University, Chicago, IL USA

## Background

Cardiac magnetic resonance (CMR) imaging is the reference standard for assessing biventricular systolic function. However, global measures of systolic function (i.e. ejection fraction) are relatively insensitive to regional alterations in myocardial structure, creating a need for quantitative analysis for regional myocardial function. Tissue phase mapping (TPM) is a CMR imaging technique that measures the regional myocardial velocities with high temporal resolution, complete left ventricular (LV) coverage, and full access to the 3-directional myocardial velocities. In this study, we evaluated the impact of myocardial scar detected with delayed enhancement imaging (DE-CMR) in patients with suspected infiltrative cardiomyopathy on global and regional myocardial velocities and dyssynchrony measured by TPM, comparing the results with controls.

## Methods

Cardiac MRI including DE-CMR and TPM in the short axis orientation (base, mid, apex) was performed in 49 patients (53.5 ± 15 years) with suspected infiltrative cardiomyopathy and 20 healthy aged-matched controls (52 ± 4 years). Data analysis was based on the AHA 16-segment model to assess the relationship between the presence of scar and altered LV velocities. DE-CMR images were evaluated for the presence of scar. TPM analyses included the quantification of radial and long-axis systolic and diastolic peak LV velocities and time-to-peak (TTP) velocities. Global peak velocities were calculated as the average over all 16 segments. The standard deviations of TTP across all 16 LV segments were used to evaluate the extent of myocardial dyssynchrony.

## Results

**Figure 1:**
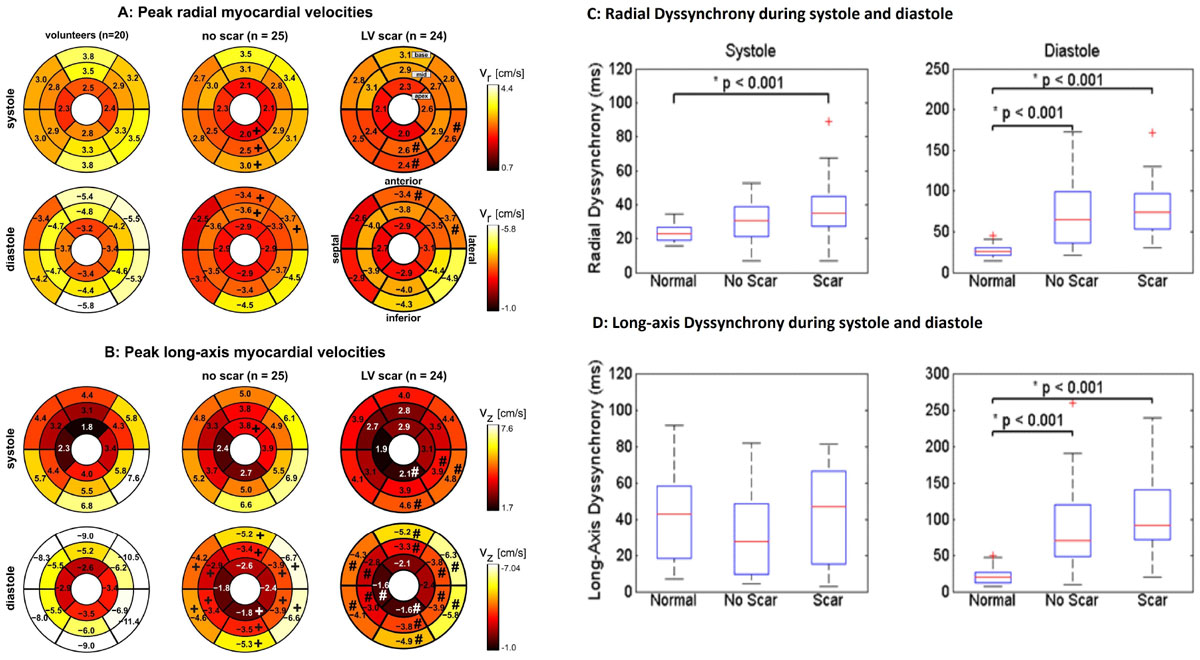
**The individual plots show the distribution of peak systolic and diastolic radial (A) and long-axis (B) myocardial velocities in the AHA 16-segment model in healthy subjects, and patients with and without scar**. # Means significant difference between patients with scar compared to age matched controls. + Means significant difference between patients with no scar compared to age matched controls. Systolic and diastolic left ventricular radial (C) and long-axis (D) dyssynchrony in healthy subjects and patients with and without scar. Increased LV dyssynchrony was observed for both diastolic and systolic LV velocities when compared to controls (P < 0.001).

DE-CMR revealed myocardial scar in 25 patients (52 ± 15 years) and no scar in 24 patients (55 ± 16 years). Both LV systolic and diastolic peak velocities were significantly decreased in patients compared with controls (P < 0.001). This was most prominent for diastolic long-axis velocities for patients with and without scar (-3.73 ± 1.29 cm/s vs. -6.54 ± 1.36 cm/s) (P < 0.001) (Table 1). The pairwise comparison of regional velocities in individual LV segments revealed generally higher systolic and diastolic peak velocities in age matched controls compared with patients (Figure 1). In patients, increased LV dyssynchrony was observed for both diastolic and systolic velocities when compared to controls (P < 0.001). LV dyssynchrony was more prominent for diastolic long-axis velocities (101.26 ± 59.9 ms vs. 21.99 ± 13.11 ms) (P < 0.001) (Figure 1, table 1). Multiple linear regression analysis showed that radial and long axis velocities were inversely associated with age, and scar burden and positively related with ejection fraction (P < 0.05).

**Table 1:** Global systolic and diastolic radial and long-axis peak velocities and dyssynchrony in patients with suspected infiltrative cardiomyopathy (n = 49) and controls (n = 20).

		Peak velocities [cm/s]		Dyssynchrony [ms]	
		Radial	long-axis	Radial	long-axis
Patients	Systole	2.67 ± 0.64	4.05 ± 2.02	34.83 ± 14.96	37.98 ± 26.29
	Diastole	-3.43 ± 0.84	-3.73 ± 1.29	75.51 ± 39.46	101.26 ± 59.89
Controls	Systole	3.07 ± 0.45*	4.54 ± 0.91	23.28 ± 4.96**	40.41 ± 24.20
	Diastole	-4.46 ± 0.87***	-6.54 ± 1.36***	27 ± 9.10***	21.99 ± 13.11***

*, **, and *** indicate P < 0.05, P < 0.01, and P < 0.001 respectively.

## Conclusions

Patients with suspected infiltrative cardiomyopathy and myocardial scar demonstrate LV dyssynchrony and significant reductions in global and regional LV velocities compared to age-matched healthy controls. Both myocardial scar and measures of global LV systolic function (i.e. ejection fraction) were independent predictors of reduced radial and long axis LV velocities.

